# The role of text messaging intervention in Inner Mongolia among patients with type 2 diabetes mellitus: a randomized controlled trial

**DOI:** 10.1186/s12911-020-01129-7

**Published:** 2020-05-14

**Authors:** Xuemei Wang, Dan Liu, Maolin Du, Ruiqi Hao, Huiqiu Zheng, Chaoli Yan

**Affiliations:** 1 Department of Health Statistics, School of Public Health, Inner Mongolia Medical University, Hohhot, China; 2 Department of Medicine, Hetao College, Bayan Nur, China; 3 Department of Endocrinology, Affiliated Hospital of Inner Mongolia Medical University, Hohhot, China

**Keywords:** Type 2 diabetes mellitus, mHealth, Randomized controlled trial

## Abstract

**Background:**

Short messages service (SMS) provides a practical medium for delivering content to address patients to adherence to self-management. The aim of study was to design some patient-centered health education messages, evaluate the feasibility of messages, and explore the effect of this model.

**Methods:**

The messages were designed by a panel of experts, and SMS Quality Evaluation Questionnaire was used to evaluate their quality. A two-arm randomized controlled trial was conducted to evaluate the effectiveness of this management model. Participants were randomly divided into an intervention group (IG) who received evaluated messages and a control group (CG) who received regular education. The primary outcomes were changes in plasma glucose and control rates, and the secondary outcomes were improvements in diet control, physical activities, weight control, etc.

**Results:**

A total of 42 messages covering five main domains: health awareness, diet control, physical activities, living habits and weight control were designed, and the average scores of the messages were 8.0 (SD 0.7), 8.5 (SD 0.6), 7.9 (SD 1.0), 8.0 (SD 0.7), and 8.4 (SD 0.9), respectively. In the SMS intervention, 171 patients with an average age of 55.1 years were involved, including 86 in the CG and 85 in the IG. At 12 months, compared with the control group (CG), the decrease of fasting plasma glucose (FPG) (1.5 vs. 0.4, *P* = 0.011) and control rate (49.4% vs. 33.3%, *P* = 0.034), the postprandial glucose (PPG) (5.8 vs. 4.2, *P* = 0.009) and control rate (57.8% vs. 33.7%, *P* = 0.002) were better in the intervention group (IG). In terms of self-management, improvements in weight control (49.3% vs. 28.2%, *P* = 0.031), vegetables consumption (87.3% vs. 29.0%, *P* < 0.001), fruits consumption (27.5% vs. 7.4%, *P* = 0.022), and physical activities (84.7% vs. 70.0%, *P* = 0.036) were better in the IG than in the CG.

**Conclusions:**

The overall quality of the messages was high. It was effective and feasible to carry out an SMS intervention to improve the behavioral habits of patients with chronic diseases in remote and undeveloped areas.

**Trial registration:**

Clinicaltrials.gov, ChiCTR1900023445. Registered May 28, 2019--Retrospectively registered.

## Background

According to the eighth edition of the Global Diabetes Map released by the International Diabetes Federation, there were 425 million adults worldwide with diabetes in 2017, and this number is expected to increase to 629 million by 2045 [1]. The prevalence of diabetes in China (11.6%) is three percentage points higher than the global prevalence, which is growing especially rapidly in low-and middle-income countries [2, 3]. Moreover, individuals with diabetes are at a higher risk of heart disease, stroke, and kidney disease, and diabetes carries a heavy financial burden for these patients’ families [4]. According to a report on the global medical expenses of patients with diabetes, China ranks second with $110 billion in 2017 [5]. Faced with such a huge expenditure, the level of glycemic control in China also does not inspire optimism, only 32.6% of adult’s plasma glucose levels have been controlled [5].

Type 2 diabetes mellitus (T2DM) and its complications can be controlled through long-term management [6, 7], but most diagnosed patients cannot adhere self-management to an extended time, especially after they left hospital. Therefore, real-time health education and reminders are essential. With the development of science and technology, the mobile phone technologies have proposed a possible solution to this problem. Some small-scale studies using information technology for diabetes management have shown the advantages for improving glycemic control [8–11]. Interventions through application (APP) and short message service (SMS) of mobile phones are common methods. APP is widely used, but the mobile phone must be a smart phone, and 4G network or Wi-Fi is required, and the coverage is relatively limited, especially in underdeveloped area. In contrast, the SMS, it is a feature of all mobile phones that receive information have the advantages of economical, convenient, real-time, and easy operation [12]. In particular, SMS intervention applies to non-smartphones, which is suitable for the economically undeveloped areas. On the other hand, while the rapid advancement of the internet has brought a lot of information and convenience to people, at the same time it has also led to inequality in access to information for different economic, educational and age groups. SMS for health education has relatively eliminated this inequality.

Inner Mongolia, a remote economically underdeveloped region, with limited medical resources, is located in northern China. Smart phones are not widespread, especially among the elderly. SMS is more suitable for Inner Mongolia. Most herders are far from medical institutions, who have a high demand for health management of the disease. According to the investigation data analysis of a surveillance survey of chronic disease and nutrition in Chinese adults in Inner Mongolia in 2015, the prevalence of diabetes is 8.75%, the treatment rate and control rate are 46.17 and 18.22%, respectively. The application of SMS in self-management not only solves the problem of shortage of medical resources, but also avoids the difficulty of face-to-face instruction caused by poor services accessibility [13].

Therefore, the objective of this study was to design a series of patient-centered health education messages for patients with T2DM, based on American Diabetes Association on Taking Care of Diabetes, following a standard process and then evaluate its effect of the management model by SMS to provide an economic and effective model for elderly diabetes management in poor areas.

## Methods

### Study design

This was a randomized, single-blind (only the researcher knows the grouping of patients), parallel-group, controlled trial. The study included two aspects, the first was the SMS design, involving the SMS quality evaluation, conducted from October 2016 to April 2017. The second was the SMS intervention, which lasted for 12 months and conducted from May 2017 to April 2018.

### Patient population

Patients with T2DM were all admitted to the Department of Endocrinology at the Affiliated Hospital of Inner Mongolia Medical University from October 2016 to March 2017 were selected for participation in the study.

Based on the results of a previous study [14], we expected an effect size of 0.26 in the plasma glucose level between baseline and at the end of intervention. The sample size was estimated with 80% power and a two-sided significance level of *α* = 0.05. We estimated a 10% dropout rate in the follow-up because of inconvenience or the time-consuming nature of the trial. We calculated a minimum sample size of 73 participants in each group.

The inclusion criteria for participants in the SMS quality evaluation were willingness and ability to participate in the SMS quality evaluation. Exclusion criteria were severe illness or inability to complete the SMS assessment.

For SMS intervention research participants, the inclusion criteria were as follows:

1. Diagnose with T2DM, and aged ≥18 years;
2. Participants or a family member who lived together were literate and had a mobile phone;
3. Clear consciousness and normal thinking; and
4. Provided informed consent for the study.

The exclusion criteria for participants in the SMS intervention were as follows:

1. Inability to complete the questionnaire because of serious illness; or
2. Participants and their relatives had a lower level of education and could not read text messages.

The study protocol was approved by the Biomedical Research Ethics Committee of Inner Mongolia Medical University, and all selected participants signed informed consent form. Over the course of the study, staff members protected the personal information of participants. Subjects were free to withdraw, and the researchers ensured that the participants’ rights were not affected.

### Data collection

Data were collected at baseline using the Diabetes Questionnaire, which was designed based on the Chinese Adult Chronic Diseases and Nutrition Monitoring Personal Questionnaire (2015). The content mainly included socio-demographic data, health-related behaviors, disease status, and dietary conditions, etc. And biochemical indicators, including fasting plasma glucose (FPG), postprandial glucose (PPG), glycosylated hemoglobin (HbA1c), Total cholesterol, triglyceride, high density lipoprotein-c (HDL-C), and low density lipoprotein-c (LDL-C), were assessed using discharge data.

### Design and methodology of SMS

#### SMS design

The messages were designed by a panel of experts, including endocrinology, chronic disease management, health education, disease prevention, etc. A total of 42 messages covering five main domains: health awareness, diet control, physical activities, living habits and weight control were designed, respectively. The content of messages was based on the report of the American Diabetes Association on Taking Care of Diabetes [15]. In terms of health awareness, we considered that participants need to have a full understanding of the etiology, process, and complications of diabetes, which is beneficial to the plasma glucose control. For diet control, this was considered to be the most direct factor affecting glycemic control [16, 17]. The diet in this study included intake of vegetables, fruits, salt, protein, fat and food, and a low glycemic index, especially for cooking and eating habits. In terms of physical activity and weight control, as we all known that patients with T2DM can effectively reduce plasma glucose and lose weight through exercise. Therefore, we helped patients choose the best mode of exercise, and ensure the best time and frequency of exercise through SMS intervention. Smoking and drinking are almost all risk factors for chronic diseases including diabetes. Studies have shown that plasma glucose can be controlled by quitting smoking and limiting alcohol consumption [18]. Therefore, we intervened on unhealthy living habits. Each text message covered only one topic, and contained 70 words or fewer. The language expression was simple, direct, and easy to understand. Final list of SMS was shown in Additional file 1.

Message design was based on the Trans-Theoretical Model (TTM) [19], which is a widely used theory to promote health behavior. For diabetic patients, the pre-contemplation stage is almost non-existent, and combined with the SMS quality evaluation, we directly intervened from the contemplation stage. Therefore, in our study, the intervention was divided into four stages: the contemplation stage, the preparation stage, the action stage, and the reinforcement stage. The contemplation stage focused on education about basic knowledge about diabetes. This stage aimed to make participants aware of the importance of integrated diabetes management and to develop awareness of health management. The preparation and action stages focused on providing tips and suggestions for changing behaviors and on guiding participants to gradually develop the attitudes and actions necessary to change their behaviors. The reinforcement stage aimed to consolidate the changes in behaviors and transform healthy behaviors into habits. Examples of message in different stages were shown in Additional file 2.

#### SMS quality evaluation

The overall quality of short message was assessed using the SMS Quality Assessment Questionnaire, which included three aspects: the understanding of the text messages, the willingness to act on the text messages, and the current status of the response to the text messages. Specifically, five questions were asked for regarding each short message: Questions 1 and 2 reflected the participant’s understanding of the text message (yes = 1, no = 0), questions 3 and 4 reflected the participant’s willingness to act on the text message (yes = 2, no = 0), and question 5 reflected the status of the participant’s response to the text message (known and have done = 1, unknown but have done = 2, known but not have done = 3, unknown and not have done = 4). The setting of the score was based on the principle of scoring the scale and the patients’ demand for message, the higher the demand, the higher the score setting [20]. The total score of each message was calculated as the sum of the scores of the five questions, reflecting the overall quality of the message, with a maximum total of 10 points. We classified > 7 as high quality, 4–7 as medium quality and < 4 as low quality. The SMS Quality Assessment Questionnaire was shown in Additional file 3.

#### SMS intervention

The intervention period was from May 2016 to May 2017. A total of 171 discharged participants with diabetes were randomly divided into two groups using random number. Participants in the intervention group (IG, 85) received evaluated messages, which were sent twice a week, only one message at a time. The content of the text messages during the same stage included five different domains. Combined with the SMS evaluation results, for some text message, more than 60% of participants reported that they did not take action, we added the transmission frequency and repeated every 4 weeks. Simultaneously, in order to reduce the rate of loss to follow-up, participants in the control group (CG, 86) were sent regular messages, mostly limited to general theoretical knowledge. Examples of regular message that CG received are shown in Additional file 4.

Telephone follow-up after each stage of the intervention was conducted. The purpose of the telephone follow-up was to ensure participants compliance and thus to ensure the quality of the study. The follow-up questionnaire was shown in Additional file 5.

### Outcomes and measurement

The main outcomes were the changes of plasma glucose and the control rate of each index. The Guidelines for the Prevention and Treatment of Type 2 Diabetes in China (2017 edition) [2], defines the FPG control rate as the proportion of all managed participants with FPG from 4.4 to 7.0 mmol/L and PPG control rate as participants with PPG < 10.0 mmol/L as a percentage of all managed participants. The secondary outcomes were changes in diet control (vegetables and fruits consumption), physical activities (more than 30 min per exercise, three to five times per week), living habits (smoking and drinking) and weight control. Each outcome measure was obtained by telephone follow-up.

### Statistical analysis

Data were recorded using EpiData 3.1 software. Data analysis was performed using IBM SPSS Statistics, Version 19.0 (IBM Corp, Armonk, NY, USA) software. Continuous variables were expressed as means (standard deviation, SD), and *t***-**test was used for comparisons between the two groups. Categorical variables were expressed as percentages, and *χ*^*2*^ test was used for comparisons between the two groups. *P* ≤ 0.05 was considered statistically significant.

To compare the health behavior of the two groups over time, we converted the behavior change including vegetable and fruit intake, weight control, and physical activities into score at baseline and 12 months. Each instance of behavior change from healthy to unhealthy status was assigned a score of − 1, change from unhealthy to healthy status was assigned a score of 1, and no change was assigned a score of 0. The scores for each of the four behaviors were then summed, generating a composite change score that ranged from − 4 (when all four behaviors changed from healthy to unhealthy) to 4 (when all four behaviors changed from unhealthy to healthy after the intervention). We then compared the difference in total scores between the two groups.

## Results

**Fig. 1:**
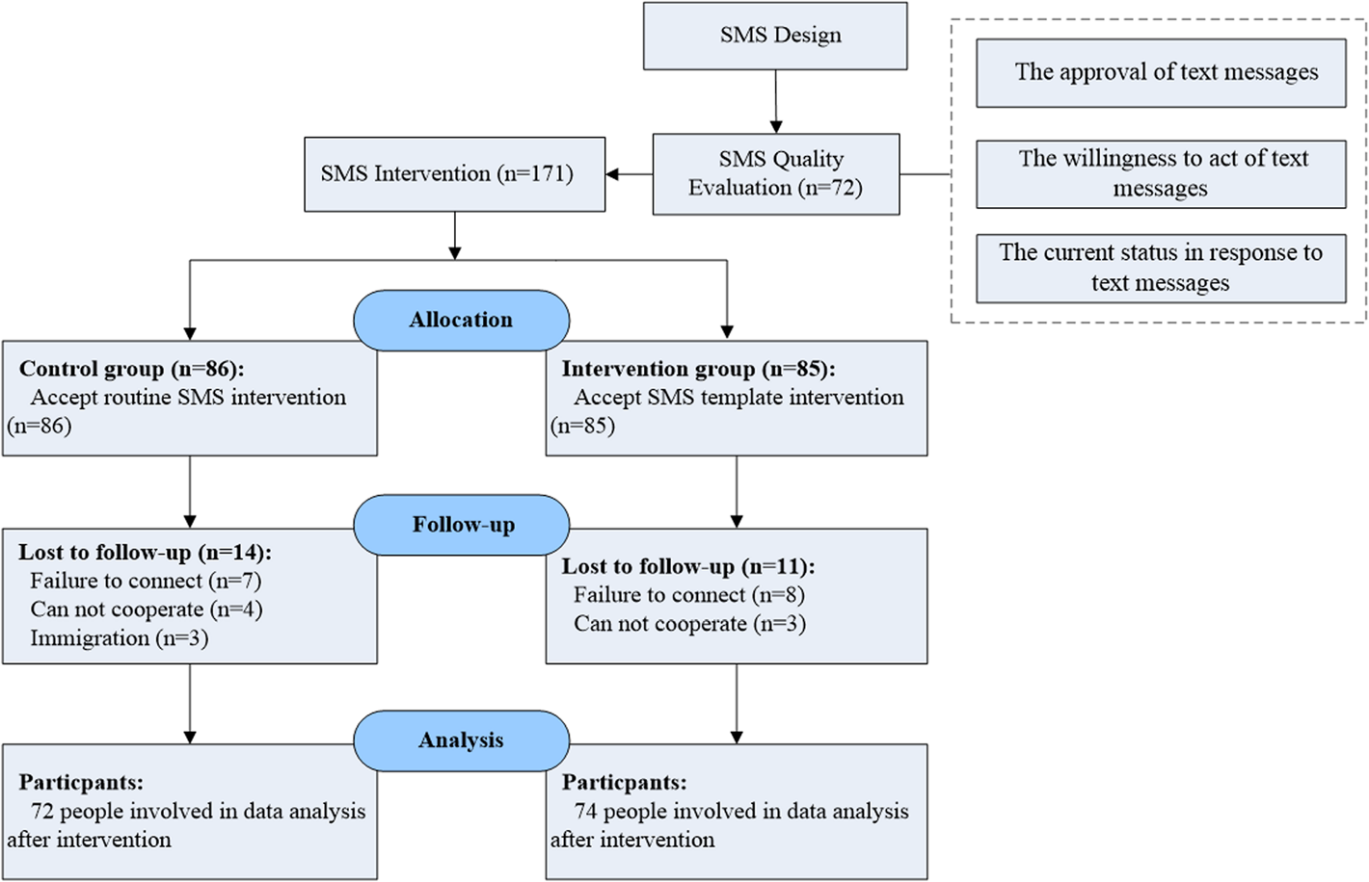
Study flow diagram

A flow diagram of the participant selection process was shown in Fig. 1. A total of 72 were selected to complete the SMS quality evaluation questionnaire. And 171 eligible participants were eventually enrolled in the SMS intervention, a total of 146 participants completed the one-year intervention to the end of the follow-up period.

### SMS quality evaluation

Table 1 showed the characteristics of the SMS quality evaluation population and the SMS intervention population. There were no significant differences in age, gender, body mass index (BMI), urban and rural, residence, education, and smoking, drinking, or hypertension (all *P* > 0.05).

**Table 1:** Comparison the characteristics of the SMS quality evaluation population and the SMS intervention population

	SMS intervention populations(*N* = 171)	SMS quality evaluation population (*N* = 72)	*χ*^2^	*P* value
Age			0.001	0.982
< 50	52 (30.4)	22 (30.6)		
≥ 50	119 (69.6)	50 (69.4)		
Gender			0.400	0.527
male	97 (56.7)	44 (61.1)		
female	74 (43.3)	28 (38.9)		
BMI				0.761 ^a^
< 18.5	4 (2.4)	3 (3.7)		
18.5 ~ 24.0	44 (26.3)	19 (23.2)		
≥ 24.0	119 (71.3)	60 (73.2)		
Distribution			0.957	0.328
urban	133 (77.8)	60 (83.3)		
rural	38 (22.2)	12 (16.7)		
Education			0.412	0.521
below high school	68 (40.0)	32 (44.4)		
high school and above	102 (60.0)	40 (55.6)		
Marital status				0.773 ^a^
married	161 (94.2)	67 (93.1)		
other	10 (5.8)	5 (6.9)		
Employment status			2.605	0.106
employed	90 (52.6)	46 (63.9)		
non-employed	81 (47.4)	26 (36.1)		
Smoking			0.011	0.915
yes	44 (25.7)	19 (26.4)		
no	127 (74.3)	53 (73.6)		
Drinking			1.317	0.251
yes	58 (33.9)	30 (41.7)		
no	113 (66.1)	42 (58.3)		
Hypertension			0.262	0.609
yes	65 (39.6)	26 (36.1)		
no	99 (60.4)	46 (63.9)		

*SMS* short message service, *BMI* body mass index. ^a^ Fisher probabilities method

Table 2 showed the results for the overall quality of the text messages. The average scores for health awareness, diet control, physical activities, living habits, and weight control were 8.0 (SD 0.7), 8.5 (SD 0.6), 7.9 (SD 1.0), 8.0 (SD 0.7), and 8.4 (SD 0.9), respectively.

**Table 2:** Comprehensive quality of the message content (%)

Main domain	Average score (SD)	Maximum score	Minimum score
Health awareness	8.0 (0.7)	9.6	6.6
Diet control	8.5 (0.6)	9.8	6.8
Physical activities	7.9 (1.0)	10.0	6.8
Living habits	8.0 (0.7)	9.2	6.8
Weight control	8.4 (0.9)	10.0	5.5

Table 3 showed the result of the participants’ understanding of the message content. The participants who completed the SMS quality evaluation, more than 98.0% believed that all kinds of text messages were understandable and approved.

**Table 3:** Participants’ understanding of the message content (%)

Main domain	understandable and approved	cannot understand but approved	understandable but disapproved	cannot understand and approved
Health awareness	99.8	0.0	0.2	0.0
Diet control	99.9	0.0	0.1	0.0
Physical activities	99.7	0.0	0.3	0.0
Living habits	98.5	0.0	1.5	0.0
Weight control	99.5	0.0	0.5	0.0

Table 4 showed the results of the participants’ willingness to act on the message content. Of the participants who completed the SMS quality evaluation, 90.5% indicated they were willing to act on the diet control messages and that they found it helpful and can be persistent, 68.3% indicated they were willing to act on the living habits messages and that they found it helpful and can be persistent.

**Table 4:** Participants’ willingness to act on the message content (%)

Main domain	Helpful and persistent	Unhelpful but persistent	Helpful but cannot insist	Unhelpful and cannot insist
Health awareness	90.1	1.8	7.3	0.8
Diet control	90.5	1.8	5.8	1.8
Physical activities	80.8	1.6	7.3	10.3
Living habits	68.3	1.4	26.2	4.4
Weight control	81.3	1.8	12.7	4.2

Table 5 showed the results of the participants’ current status in response to the text messages. The participants who completed the SMS quality evaluation, 79.8% believed that the living habits messages were known but who reported not having taken action.

**Table 5:** Current status of the participant in response to the text message content (%)

Main domain	Known and have done	Unknown but have done	Known but not have done	Unknown and not have done
Health awareness	44.6	2.4	36.7	16.3
Diet control	29.6	7.0	20.8	42.5
Physical activities	40.4	6.8	17.2	35.7
Living habits	8.7	1.6	79.8	9.9
Weight control	28.5	6.7	20.1	44.7

### SMS intervention effect evaluation

Table 6 showed there were no statistical differences in baseline characteristics between the two groups (all *P* < 0.05). The participants were 97 males and 74 females. The average age for the total population was 55.1 (SD 10.8) years. The average level of FPG was 9.0 (SD 2.6), and the average level of PPG was 16.6 (SD 4.4). The control rates of FPG and PPG were 28.4% (48/171), 9.6% (16/171), respectively. 97.6% (80/85) in the IG and 90.5% (76/86) in the CG were receiving hypoglycemic therapy. And 36.5% (31/85) in the IG and 33.7% (29/86) in the CG were receiving antihypertensive therapy.

**Table 6:** Baseline characteristics of the participants in the intervention group and control group

	Total	IG (*n* = 85)	CG (*n* = 86)	*t/χ*^2^	*P* value
Demographic, Mean (SD)					
Gender (Male/Female)	97/74	42/43	55/31	3.682	0.055
Age (years)	55.1 (10.8)	55.4 (9.7)	54.7 (11.8)	−0.416	0.678
BMI (Kg/m^2^)	25.8 (3.8)	25.9 (3.5)	25.8 (4.1)	−0.255	0.799
Indicators, Mean (SD)					
FPG (mmol/L)	9.0 (2.6)	9.2 (2.8)	8.8 (2.4)	−0.954	0.342
PPG (mmol/L)	16.6 (4.4)	16.8 (4.6)	16.4 (4.3)	−0.599	0.550
SBP (mmHg)	132.3 (17.0)	131.0 (16.1)	134.3 (17.8)	1.137	0.257
DBP (mmHg)	80.8 (11.8)	79.8 (9.8)	81.8 (13.5)	1.034	0.303
FPG control (%)	48 (28.4)	25 (29.8)	23 (27.1)	0.152	0.697
PPG control (%)	16 (9.6)	10 (12.0)	6 (7.2)	1.107	0.293
SBP control (%)	90 (60.8)	51 (68.0)	39 (53.4)	3.298	0.069
DBP control (%)	92 (63.4)	50 (68.5)	42 (58.3)	1.613	0.204
Total cholesterol (mg/l)	4.7 (1.2)	4.8 (1.2)	4.7 (1.2)	−0.747	0.456
Triglycerides (mg/l)	1.9 (1.3)	1.9 (1.3)	1.9 (1.2)	0.275	0.783
HDL-C (mg/l)	1.1 (0.3)	1.1 (0.2)	1.1 (0.3)	−1.062	0.290
LDL-C (mg/l)	3.0 (0.9)	3.0 (0.9)	2.9 (1.0)	−0.739	0.461
HbAlC (%)	9.2 (2.0)	9.2 (2.0)	9.1 (2.0)	−0.339	0.735
Risk factors					
Hypertension (%)	65 (39.6)	32 (39.0)	33 (40.2)	0.025	0.873
Dyslipidemia (%)	96 (56.1)	46 (54.1)	50 (58.1)	0.126	0.722
Smoking (%)	44 (25.7)	23 (27.1)	21 (24.4)	0.156	0.693
Drinking (%)	58 (33.9)	26 (30.6)	32 (37.2)	0.361	0.836
Overweight (%)	97 (58.1)	50 (61.0)	47 (55.3)	0.553	0.457
Pain (%)	94 (55.3)	41 (48.2)	53 (62.4)	3.427	0.064
Anxiety (%)	82 (48.2)	43 (50.6)	39 (45.9)	0.377	0.539
Hypoglycemic therapy (%)	156 (94.0)	80 (97.6)	76 (90.5)	2.534	0.111
Medication way (%)				3.058	0.217
insulin	80 (51.9)	46 (57.5)	34 (45.9)		
Oral medication + insulin	48 (31.2)	20 (25.0)	28 (37.8)		
Oral medication	26 (16.9)	14 (17.5)	12 (16.2)		
Antihypertensive therapy (%)	60 (35.1)	31 (36.5)	29 (33.7)		0.672^†^

*CG* control group, *IG* intervention group, *SD* standard deviation, *BMI* body mass index, *FPG* fasting plasma glucose, *PPG* postprandial glucose, *SBP* systolic blood pressure, *DBP* diastolic blood pressure, *HDL-C* high density lipoprotein-c, *LDL-C* low density lipoprotein-c

^†^Fisher probabilities method

Table 7 showed changes in plasma glucose, and diabetes risk factors over the entire intervention period. At 12 months, compared with the CG, the decrease in the FPG (1.5 vs. 0.4, *P* = 0.011), and the PPG (5.8 vs. 4.2, *P* = 0.009) were better in the IG.

**Table 7:** Change in primary outcomes during the intervention

	Total		CG		IG		*t*^*b*^	*P* value
	At 12 month	Change ^a^	At 12 month	Change ^a^	At 12 month	Change ^a^		
Primary outcomes, Mean (SD)								
FPG (mmol/L)	8.0 (2.1)	−0.9 (2.9)	8.4 (2.2)	−0.4 (2.8)	7.7 (1.9)	−1.5 (3.0)	2.556	0.011^*^
PPG (mmol/L)	11.1 (2.8)	−5.4 (4.8)	11.9 (3.2)	−4.2 (4.7)	10.4 (2.2)	−5.8 (5.1)	2.633	0.009^*^

^*^P < 0.05. ^a^ change = Endpoint value – Baseline value; ^b^ The t value was the comparative result of the changes between the CG and the IG

*FPG* fasting plasma glucose, *PPG* postprandial glucose, *CG* control group, *IG* intervention group, *SD* standard deviation

Table 8 showed the results of plasma glucose control rate and health behaviors improvements at 12 month. The FPG control rate (49.4% vs. 33.3%, *P* = 0.034), and the PPG control rate (57.8% vs. 33.7%, *P* = 0.002) were better in the IG, compared with the CG. In terms of self-management, improvements in weight control (49.3% vs. 28.2%, *P* = 0.031), vegetables consumption (87.3% vs. 29.0%, *P* < 0.001), fruits consumption (27.5% vs. 7.4%, *P* = 0.022), and physical activities (84.7% vs. 70.0%, *P* = 0.036) were better in the IG than in the CG. There was no difference between the two groups in terms of quitting smoking or drinking (both *P* > 0.05).

**Table 8:** Control rate of plasma glucose and improvements of health behaviors at 12 month

	Total	CG	IG	*t/χ*^2^	*P* value
Primary outcomes					
FPG control (%)	70 (41.4)	28 (33.3)	42 (49.4)	4.501	0.034^*^
PPG control (%)	76 (45.8)	28 (33.7)	48 (57.8)	9.708	0.002^*^
Secondary outcomes					
Body Weight (%)				6.972	0.031^*^
Increased from Baseline	27 (19.0)	17 (23.9)	10 (14.1)		
Decreased from Baseline	55 (38.7)	20 (28.2)	35 (49.3)		
Unchanged from Baseline	60 (42.3)	34 (47.9)	26 (36.6)		
Vegetables consumption (%)				49.081	< 0.001^**^
Increased from Baseline	82 (58.6)	20 (29.0)	62 (87.3)		
Decreased from Baseline	19 (13.6)	16 (23.2)	3 (4.2)		
Unchanged from Baseline	39 (27.9)	33 (47.8)	6 (8.5)		
Fruits consumption (%)				7.634	0.022^*^
Increased from Baseline	15 (16.0)	4 (7.4)	11 (27.5)		
Decreased from Baseline	44 (46.8)	26 (48.1)	18 (45.0)		
Unchanged from Baseline	35 (37.2)	24 (44.4)	11 (27.5)		
Physical activities (%)				4.407	0.036^*^
Continuous exercise	110 (77.5)	49 (70.0)	61 (84.7)		
Discontinuous exercise	32 (22.5)	21 (30.0)	11 (15.3)		
Quit smoking (%)	12 (25.5)	3 (13.6)	9 (36.0)	3.078	0.079
Quit drinking (%)	17 (39.5)	9 (34.6)	8 (47.1)	0.666	0.415

^*^P < 0.05; ^**^P < 0.001

*FPG* fasting plasma glucose, *PPG* postprandial glucose, *CG* control group, *IG* intervention group

**Fig. 2:**
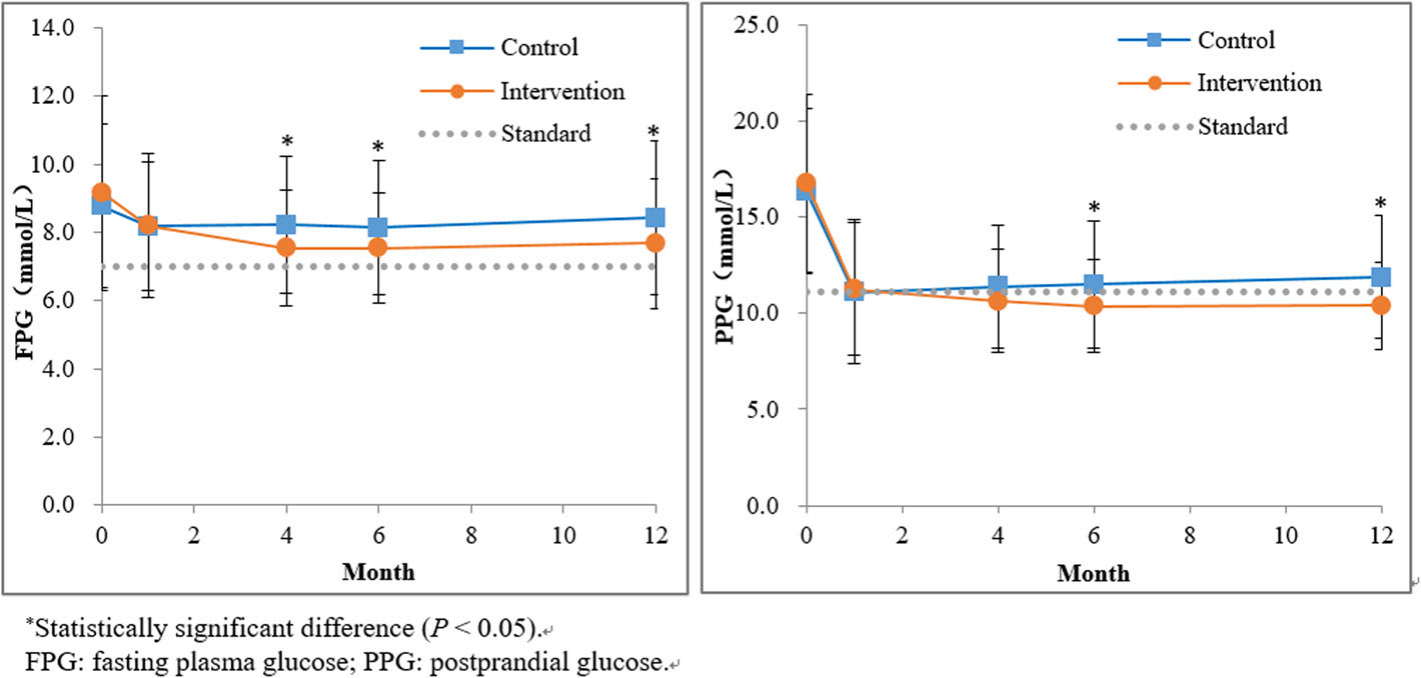
Comparison of FPG and PPG levels at the end of each intervention stage

Figure 2 showed the comparison of FPG and PPG changes between the baseline and the end of the four intervention stages. The FPG level in the IG was superior to that in the CG at the end of the preparation stage, the end of the action stage, and the end of the reinforcement stage (*P* < 0.05). The PPG level in the IG was superior to that in the CG at the end of the action stage and the end of the reinforcement stage (*P* < 0.05).

**Fig. 3:**
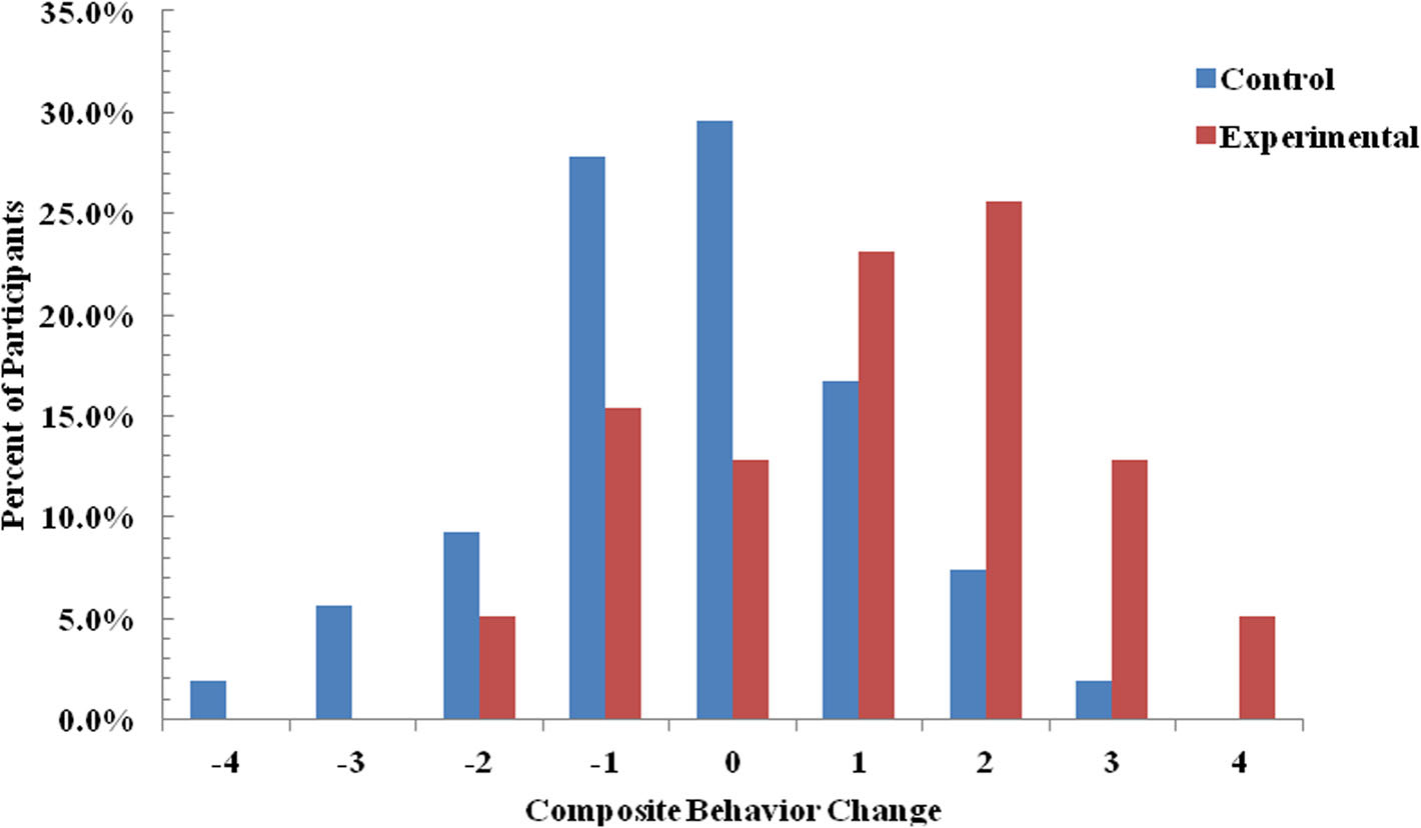
Composite change in scores by intervention group

Figure 3 showed the composition of behavior change scores for two groups. Negative scores indicated deterioration in behaviors at 12 months compared to baseline. The score of IG ≤ − 1 accounted for 20.0% (17/85), while that of CG was 44.2% (38/86). Positive scores indicated improvement in behaviors at 12 months compared to baseline. A total of 67.1% (57/85) of participants in the IG scored ≥1, while only 25.6% (22/86) in the CG. The mean of the total score was higher for the IG than for the CG (1.1 vs − 0.3, *P* < 0.001).

## Discussion

### SMS quality evaluation

Patients with T2DM need long-term self-management, especially in the community. High-quality messages not only to meet the needs of participants, but also to drive active behavior changes among diabetes and to ensure the effective of an SMS intervention on diabetes. In order to ensure the quality of messages, we made a judgment through the SMS quality evaluation questionnaire. The results showed that, all of the messages had a mean score of approximately 8 points, indicating the overall quality of the message was high. Diet control messages had the highest willingness to act, consistent with the patient’s higher awareness of diet control, and believed that diets were the most direct and effective way to glycemic control [21]. A high number of participants reported that the content of weight control and diet control messages were “unknown and not have done”. Inner Mongolia lacks fresh vegetables and fruits due to its remote location, and it is a minority community containing 49 ethnic groups, people lived there like wine and meat. Especially for elderly, who knew very little about the glycemic index of food, and had a fuzzy choice of vegetables and fruits. In addition, patients’ self-management was limited by medical resources and education [22]. The proportion of participants who reported not having taken action on diet control, living habits, and weight control messages were relatively high, which were consistent with their lower willingness to act on the one hand. Although participants have a certain understanding of glycemic control, they may also lack systematic theoretical knowledge or fail to establish corresponding healthy behaviors. Health beliefs and behaviors establishment must be based on correct knowledge of diabetes. Without this knowledge, the importance of glycemic control cannot be translated into behavioral improvement, but only into conception. Especially for middle-aged and elderly patients, it takes a process to completely change the bad habits they have cultivated over the years. Therefore, we need to carry out comprehensive systemic interventions to encourage patients to increase their knowledge regarding treatment attitudes and then expand this to changes in behavior.

### SMS intervention effect evaluation

The SMS intervention successfully improved participants’ continuous exercise and weight control, which was similar to previous results [23]. Sustained message alerts can increase participants subjective initiative [24]. Some participants reported that, SMS served as a reminder to guide them to choose appropriate activities and methods, it will gradually develop the habit of continuous exercise and help control weight. The SMS messages contained a large amount of dietary information, covering all aspects of a balanced diet, which guided participants to choose foods that were beneficial to glycemic control. SMS intervention did not affect smoking cessation or alcohol restriction, which was consistent with previous research [25–27]. An in-depth analysis showed that 18.2% (8/44) of smokers in this study said they would quit but not act within 1 year. In addition, 36.4% (16/44) said that they did not want to quit smoking. Among the drinkers, 79.6% (43/54) were employed, and they said drinking is a social necessity. Therefore, society and the media need to take measures to control tobacco and alcohol. Consistent with a study in India [28], the SMS intervention group had a greater improvement in awareness of diabetes risk factors. Participants had high adherence when health care professionals were able to use mobile phones to maintain close contact with participants to provide individualized education and management [29]. It has been observed that Behavior change was a gradual, phased, and complex process, which was characterized by jumps or reversals in between periods of progress [30], suggesting that we have to implement long patients behavioral intervention to promote the transformation of patient behavior.

The successful reductions in FPG and PPG levels observed in our study can be attributed to the synergistic effects of improved health behaviors. Participants might be unaware of the conditions or factors that aggravate plasma glucose levels. Previous studies have shown [31] that better self-management behavior among patients with diabetes is associated with better glycemic control. Participants in the IG had increased knowledge of the etiology, development process, complications, and influencing factors of diabetes—a first step in the process of glycemic control. Limited knowledge about diabetes resulted in poorer protective practices against the disease [32]. Diabetes and hypertension are lifestyle diseases. Hypertension is the most common complication of diabetes [33], and may worsen the condition of patients with diabetes [34]. Therefore, because of the common risk factors between two diseases, significant content on the management of BP have been added to the design of SMS messages, and for a long time, effective diabetes management can also promote stability of BP.

We found that the levels of FPG and PPG were different between the two groups after the second and third stages by comparing of the IG and CG at each intervention stage. This finding demonstrated the effectiveness of the application of the TTM, which provided targeted intervention education based on the physical activities intentions and behavioral characteristics of participants at different stages. The contemplation stage aimed to impart knowledge to the participants, the preparation stage prompted participants to elevate their knowledge to therapeutic attitudes and to develop plans to change their behaviors, and the action stage aimed to further elevate therapeutic attitudes to behavior changes. The goal was to gradually improve the participants’ multiple objective physiological indicators and reduce the risk of recurrence.

## Conclusions

The overall quality of the SMS content we designed was high enough to meet the needs of participants. Our findings support the feasibility, acceptability, and preliminary effectiveness of a low-cost, low-burden text messaging intervention to promote the effective control of plasma glucose and to prevent the degradation of health behaviors over time. This model has the potential to significantly reduce the required medical resources and to be widely popularized in economically backward areas.

### Limitations

The sample size of this study needs to be further expanded, and considering that we only selected hospitalized patients, we will select some community patient to expand the sample size in the next step. In addition, the main outcome indicators were FBG and PBG, but owing to HbA1C is the gold standard for measurement for the assessment of glycemic control, which can effectively reflect the situation over the past 1–2 months blood sugar control in diabetics, we will consider HbA1C as the main outcome indicator in the next study.

## Supplementary information

**Additional file 1.** Final list of SMS.

**Additional file 2.** Stages of change, type of content, and number of SMS text messages per key domain.

**Additional file 3.** SMS evaluation questionnaire.

**Additional file 4.** Examples of regular messages.

**Additional file 5.** Follow-up record sheet.

## Data Availability

The datasets used and/or analyzed during this study are available from the corresponding author upon reasonable request.

## Abbreviations

T2DM: Type 2 diabetes mellitus
SMS: Short message service
IG: Intervention group
CG: Control group
SD: Standard deviation
BP: Blood pressure
FBG: Fasting blood glucose
PBG: Postprandial blood glucose
SBP: Systolic blood pressure
DBP: Diastolic blood pressure
